# Concurrent Takayasu Arteritis and Microscopic Polyangiitis in a Young Female: A Complex Case of Large- and Small-Vessel Vasculitis

**DOI:** 10.7759/cureus.75833

**Published:** 2024-12-16

**Authors:** Omar Abdelhalim, Utsow Saha, Gowri Swaminathan, Zara Bhutta, Nuha Al-Howthi, Hazem Abosheaishaa

**Affiliations:** 1 Internal Medicine, Icahn School of Medicine at Mount Sinai, Queens Hospital Center, Jamaica, USA; 2 Internal Medicine/Gastroenterology, Cairo University, Cairo, EGY

**Keywords:** concurrent vasculitis, large vessel vasculitis, microscopic polyangiitis, small vessel vasculitis, takayasu arteritis

## Abstract

Vasculitides represent a range of disorders marked by inflammation of blood vessels, often posing significant diagnostic challenges due to their diverse clinical presentations and involvement of multiple organ systems. We present the case of a 26-year-old woman who arrived with hemoptysis and a background of exertional dyspnea, chest pain, and occasional visual disturbances. Initial investigations showed elevated perinuclear anti-neutrophil cytoplasmic antibodies (P-ANCAs) and myeloperoxidase antibodies (MPOs), indicative of microscopic polyangiitis (MPA). Imaging revealed circumferential thickening of the aorta and its branches, occlusion of the left subclavian and common carotid arteries, and ground-glass opacities in the lungs, suggesting the involvement of both large and small vessels.

Although the findings suggested both MPA and Takayasu arteritis (TA), differentiating between these conditions was challenging due to overlapping clinical and radiological features. Treatment with prednisone and rituximab initially brought the symptoms under control, but the patient later experienced a relapse, illustrating the complexity of managing simultaneous small- and large-vessel vasculitis. This case highlights the necessity for a comprehensive diagnostic approach and personalized treatment strategies in handling complex vasculitides with multisystem involvement.

## Introduction

Vasculitis comprises a heterogeneous group of disorders characterized by inflammation and necrosis of blood vessel walls, which can involve vessels of varying sizes and anatomical distributions. The clinical presentation of vasculitis is diverse, ranging from isolated organ involvement to multisystemic manifestations, posing significant diagnostic and therapeutic challenges [1]. Here, we present a case of a 26-year-old female with an uncommon presentation of concurrent involvement of small and large vessels in antineutrophil cytoplasmic antibody (ANCA)-associated vasculitis. 

The overlapping features of Takayasu arteritis (TA) and microscopic polyangiitis (MPA) in this patient illustrate the complexities of distinguishing between different vasculitic syndromes. This case underscores the importance of a comprehensive diagnostic approach and tailored therapeutic strategies in managing such complex presentations of vasculitis [2].

In this report, we detail the clinical course, diagnostic workup, treatment regimen, and challenges encountered in managing this rare manifestation of vasculitis. By highlighting this case, we aim to contribute to the understanding and recognition of atypical presentations of vasculitic disorders, emphasizing the need for continued vigilance and individualized care in clinical practice.

## Case presentation

**Figure 1:**
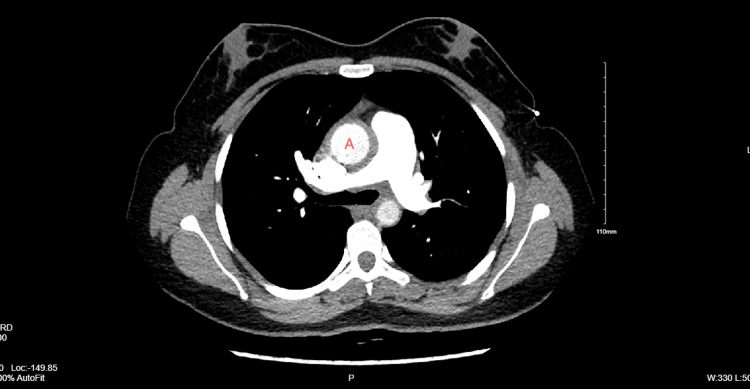
Circumferential thickening of the thoracic aorta (marked as A) and the abdominal aorta.

**Figure 2:**
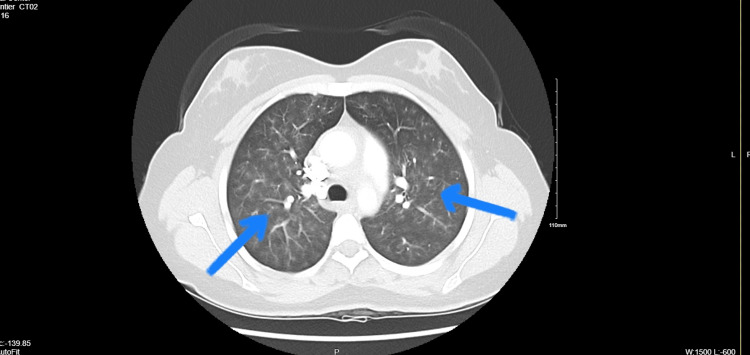
CT chest showing diffuse centrilobular bilateral ground-glass (blue arrows) opacities suggestive of inflammatory vasculitis.

A 26-year-old female was admitted to the hospital with a constellation of symptoms, including exertional dyspnea, decreased exercise tolerance, chest pain, headaches with transient blurry vision, pulsatile bilateral tinnitus, and a recent miscarriage. Given her gender, systemic symptoms, and recent miscarriage, rheumatological diseases were top differentials, prompting an extensive rheumatologic workup. This included tests such as erythrocyte sedimentation rate (ESR), C-reactive protein (CRP), cardiolipin antibodies, and β2 glycoprotein, which were negative despite markedly elevated antinuclear antibody (ANA) titers with negative anti-dsDNA. Both HIV and syphilis tests were also negative. Elevated perinuclear anti-neutrophil cytoplasmic antibodies (P-ANCA) levels and mildly elevated myeloperoxidase antibodies were noted, along with mild elevations in immunoglobulin A (IgA), IgG, and serum kappa and lambda proteins indicative of polyclonal gammopathy. Imaging studies, including CT chest and computed tomography angiography (CTA), revealed circumferential thickening of the thoracic and abdominal aorta (Figure 1), involvement of the suprarenal and renal branches, near occlusion of the left common carotid artery, complete occlusion of the left subclavian artery, and diffuse centrilobular ground-glass opacities (Figure 2), suggestive of inflammatory vasculitis.

**Figure 3:**
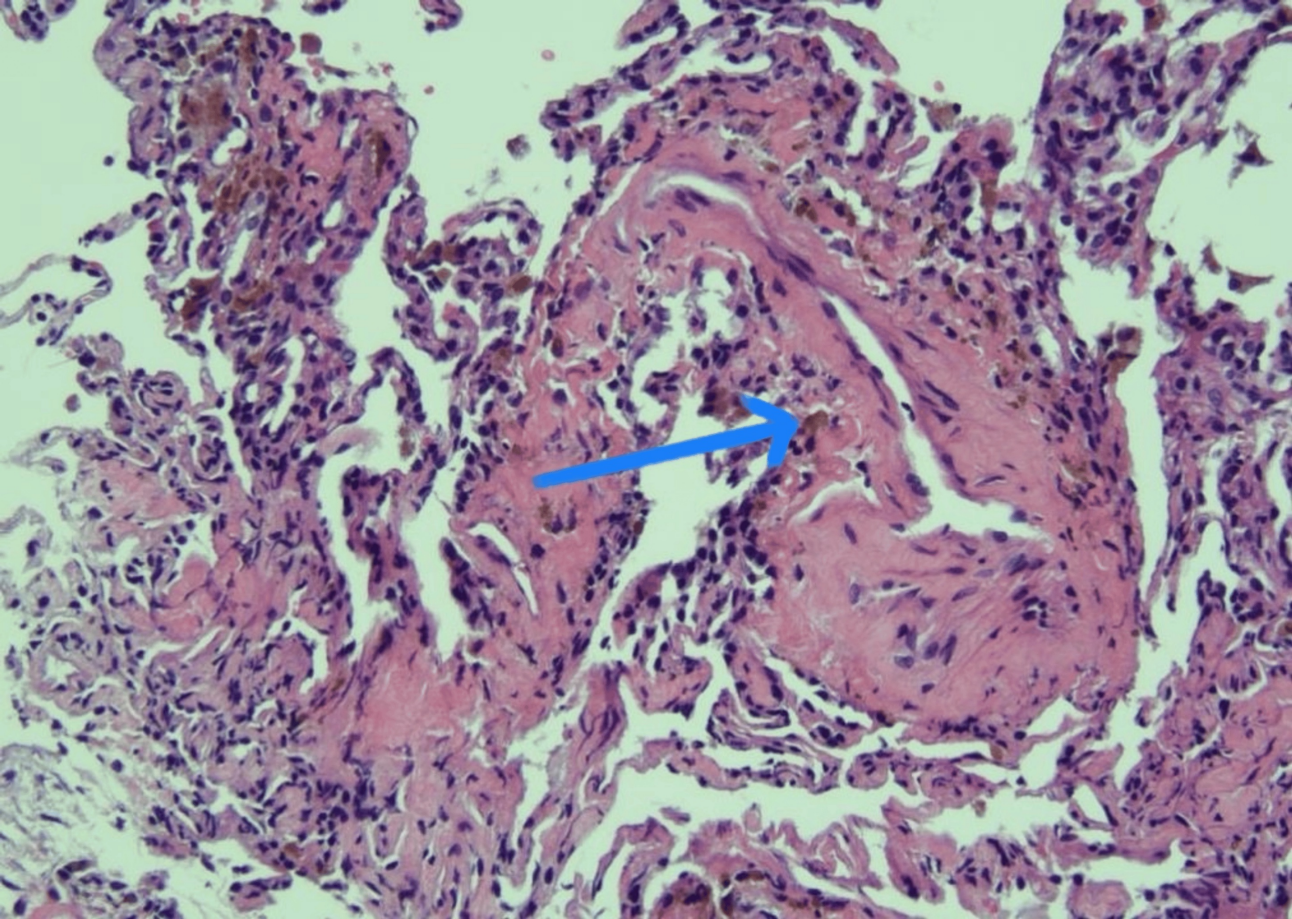
Histology (x20) showing hemosiderin-laden macrophages (blue arrow) and an increase in neutrophils around blood vessels raising suspicion for microscopic polyangiitis (MPA).

MR angiograms further confirmed the diagnosis of vasculitis. A lung biopsy via video-assisted thoracic surgery (VATS) revealed hemosiderin-laden macrophages and an increase in neutrophils around blood vessels (Figure 3), raising suspicion for MPA. Despite the initial impression, the patient's clinical presentation was complex, making it challenging to distinguish between different forms of vasculitis. Given the presence of both small and large-vessel involvement, TA was strongly considered over fibromuscular dysplasia (FMD) due to elevated inflammatory markers, which are typically not associated with FMD. Treatment with a prednisone taper and rituximab was initiated, resulting in an improvement in inflammatory markers and symptoms. However, the patient was noncompliant with medication and lost to follow-up. Six months later, she returned to the emergency room (ER) with worsening dyspnea, chest pain, and hemoptysis. Imaging showed bilateral centrilobular ground-glass opacities and worsening vasculitis with arterial stenosis and occlusion. Examination revealed weaker pulses on the left side and a systolic murmur, indicating disease progression. Laboratory tests showed chronic anemia, a negative ANA screen (previously highly positive), negative C-ANCA with positive P-ANCA, elevated myeloperoxidase antibodies, normal complement levels, and improved inflammatory markers with steroid initiation. Additional tests for autoimmune/vasculitis-related antibody markers (Table 1), infection panel, and general blood work panel were negative (Table 2).

**Table 1:** Autoimmune/vasculitis-related antibody markers. Ab, antibody; dsDNA, double-stranded DNA; EIA, enzyme immunoassay; CCP, cyclic citrullinated peptide; DRVVT, dilute Russell's viper venom time; ANCA, antineutrophil cytoplasmic antibodies; EJ, eukaryotic initiation factor; OJ, antibody against isoleucyl-tRNA synthetase; SRP, signal recognition particle; MI, nucleosome remodeling deacetylase complex protein; RNP, ribonucleoprotein; CADM, clinically amyopathic dermatomyositis; NXP-2, nuclear matrix protein 2; TIF, transcription intermediary factor 1-gamma; SS, Sjogren syndrome; SAE, small ubiquitin-like modifier activating enzyme

Test	Result	Reference range
Antinuclear Ab (ANA)	1:160	<1:80
Anti-dsDNA Ab, EIA	<12	≤29 IU/mL
CCP Ab IgG EIA	<8	≤19 Units
Rheumatoid factor	<10	≤14 IU/mL
Silica clotting time	0.80	0.00-1.16 ratio
DRVVT Interpretation	LA NEG	LA NEG
Cytoplasmic (C-ANCA) Ab	Negative	Negative
Perinuclear (P-ANCA) Ab	Positive	Negative
P-ANCA titer	>1:1280	<1:20
Sjogren's SS-A AB (RO)	<0.2	≤0.9 AI
Sjogren's SS-B AB (LA)	<0.2	≤0.9 AI
ENA - SM (Smith) AB	<0.2	≤0.9 AI
ENA - RNP AB	<0.2	≤0.9 AI
Proteinase 3 Ab, EIA	5	≤20.0 units
Proteinase 3 Ab	Negative	Negative
Myeloperoxidase Ab	31.2	≤20.0 units
Myeloperoxidase Ab	Positive	Negative
C3 complement	134	81-157 mg/dL
C4 complement	22	13-39 mg/dL
Anti-Jo-1 Ab	Negative	<20 units
PL-7 Plus	Negative	Negative
PL-12 Plus	Negative	Negative
EJ MyoMarker3 Plus	Negative	Negative
OJ MyoMarker3 Plus	Negative	Negative
SRP MyoMarker3 Plus	Negative	Negative
MI-2 Plus	Negative	Negative
Fibrillarin (U3 RNP) Plus	Negative	Negative
MDA5 (P140)(CADM-140) Plus	Negative	<20 units
NXP-2 (P140) MyoPlus	Negative	<20 units
TIF GAMMA (P155/140) Plus	Negative	<20 units
Anti-PM-Scl-100 Plus	Negative	<20 units
U2 snRNP Plus	Negative	Negative
Anti-U1-RNP Ab Plus	Negative	<20 units
Ku MyoMarker3 Plus	Negative	Negative
Anti-SS-A 52 kD Ab, IgG Plus	Negative	<20 units
Anti-SAE 1 IgG	Negative	<20 units

**Table 2:** General blood work panel. WBC, white blood cell; HgB, hemoglobin; PLT, platelet; BNP, brain natriuretic peptide; ALK PHOS, alkaline phosphatase; ALT, alanine aminotransferase; AST, aspartate aminotransferase; UIBC, unsaturated iron binding capacity; TIBC, total iron binding capacity; ESR, erythrocyte sedimentation rate

Test	Result	Reference range
WBC	6.40 x 10³/mcL	4.80 x 10³ to 10.80 x10³/mcL
HgB	10.1 g/dL	12.0-16.0 g/dL
PLT	390 x 10³/mcL	150 x 10³ to 450 x10³/mcL
Magnesium	2.5 mg/dL	1.60-2.60 mg/dL
Troponin T	<0.010 ng/mL	≤0.010 ng/mL
Pro-BNP	18 pg/mL	1-125 pg/mL
ALK PHOS	115 U/L	35-104 U/L
ALT (SGPT)	25 U/L	0-33 U/L
AST (SGOT)	24 U/L	5-32 U/L
Creatinine	0.98 mg/dL	0.50-1.20 mg/dL
BUN	19 mg/dL	6-23 mg/dL
Sodium	137 mmol/L	136-145 mmol/L
PCO₂ venous	49 mmHg	38-41 mmHg
Lactate venous	1.2 mmol/L	0.6-1.4 mmol/L
HCO₃ venous	28 mmol/L	22-29 mmol/L
D-Dimer Quant	266 ng/mL DDU	≤230 ng/mL DDU
Folate	>20.0 ng/mL	≥4.7 ng/mL
Potassium	4.7 mmol/L	3.5-5.1 mmol/L
Vitamin B12	698 pg/mL	232-1,245 pg/mL
Iron	25 µg/dL	30-160 µg/dL
UIBC	422 µg/dL	110-370 µg/dL
TIBC	447 µg/dL	220-430 µg/dL
Thyroid-stimulating hormone	1.56 µIU/mL	0.27-4.20 µIU/mL
aPTT	37.2 seconds	25.1-36.5 seconds
Ferritin	116 ng/mL	15-150 ng/mL
Creatine kinase, total, serum	62 U/L	32-182 U/L
Interleukin-6	16.3 pg/mL	0.0-13.0 pg/mL
ESR	35 mm/hour	0-20 mm/hour
High-sensitive C-reactive protein	3.60 mg/L	
Angiotensin 1 converting enzyme	19 U/L	14-82 U/L
Cholesterol	146 mg/dL	≤199 mg/dL
LDL cholesterol calculated	77 mg/dL	≤99 mg/dL

The patient was restarted on a steroid taper and rituximab infusions for ongoing management, and good disease remission was achieved after continuing rituximab for two months. The patient was noted to have an improvement in quality of life thereafter.

## Discussion

The term *vasculitides* refers to rare conditions characterized by inflammation of blood vessels, which can either drive the disease process or result from an existing condition. Vasculitides may present as isolated organ disorders or involve multiple systems. Historically classified as small-, medium-, or large-vessel vasculitis, the 1994 International Chapel Hill Consensus Conference (CHCC 1994) set the initial nomenclature for these conditions [1]. However, the 2012 International CHCC revised these classifications and introduced new categories, reflecting advances in our understanding [2]. Our case presents a rare example of concurrent small- and large-vessel vasculitis in a 26-year-old female. Large-vessel vasculitides include giant cell arteritis (GCA) and TA. TA, which predominantly affects females, is rare in the United States, with an incidence of 2 to 3 cases per million annually [3-5]. ANCA-associated vasculitides (AAV) such as MPA, granulomatosis with polyangiitis (GPA), and eosinophilic granulomatosis with polyangiitis (EGPA) typically involve small vessels, though large-vessel involvement (L-AAV) is documented in a few cases [2,6,7]. The patient's presentation aligned with MPA, a small-vessel vasculitis, and TA, a large-vessel vasculitis. TA often presents with aortitis and can lead to ischemic events [8]. It is characterized by panarteritis involving all arterial layers, which eventually evolves into fibrosis [9]. MPA commonly affects capillaries, venules, and arterioles, often manifesting as necrotizing glomerulonephritis or pulmonary capillaritis, with ANCA positivity in over 90% of cases [10,11]. Diagnostic differentiation between TA and GCA involves assessing aortic wall thickness and scarring patterns. MPA, unlike GPA, lacks granulomatous inflammation but may show rare granulomas [12]. Chirinos et al. suggested that large-vessel involvement could be part of the AAV spectrum rather than a separate condition, with GPA and MPA showing the most significant large-vessel involvement [13]. In our case, imaging and biopsy revealed features consistent with TA and possible MPA with alveolar hemorrhage. Diagnosis of vasculitides requires high suspicion due to vague, multisystem symptoms. Unexplained ischemia or multisystem disease with systemic inflammation should prompt consideration of vasculitis [14]. Our case exemplifies the challenge of diagnosing concurrent TA and AAV with both large- and small-vessel involvement. Cases of concurrent large- and small-vessel vasculitis are rare but documented. Chirinos et al. reported a case involving both AAV and TA, highlighting diagnostic difficulties [13]. Saito et al. described a case of coexisting MPA and TA, emphasizing the need for comprehensive evaluation [15]. Glucocorticoid therapy is the primary treatment for TA, often combined with non-biologic agents like methotrexate due to relapse risks [16]. Rituximab, as shown in the Rituximab in ANCA-Associated Vasculitis (RAVE) trial, is effective for AAV, with remission rates similar to cyclophosphamide but superior at six months [17]. Our patient was treated with a prednisone taper and Rituximab, resulting in gradual symptomatic improvement and sustained remission. Most patients achieve remission within three to six months, but treatment is often continued for up to six months for sustained control. In our case, the patient was continued on Rituximab for two months before achieving remission.

## Conclusions

In conclusion, this case of concurrent small- and large-vessel vasculitis in a young female underscores the diagnostic challenges and therapeutic complexities inherent in managing vasculitides with diverse clinical presentations. The overlapping features of TA and MPA in our patient exemplify the importance of comprehensive diagnostic evaluations, including imaging and histopathological studies, to delineate distinct vasculitic syndromes. Treatment strategies involving corticosteroids and Rituximab initially provided symptomatic relief but necessitated ongoing management due to disease relapse, highlighting the chronic and unpredictable nature of these conditions. This case underscores the need for continued research to optimize therapeutic approaches tailored to individualized patient profiles, aiming for sustained remission while minimizing treatment-related complications in complex vasculitic presentations.
